# Current News

**Published:** 1892-09

**Authors:** 


### Current News.

### NEW JERSEY EXAMINATIONS.

The next meeting of the New Jersey State Board of Examiners will be held at the Commission Rooms, No. 88 Broad Street, Elizabeth, New Jersey, commencing on Tuesday, October 18, 1892. Candidates will please file their applications with the Secretary before October 4. Blanks and information furnished on application.

G. Carleton Brown,
*Secretary.*

88 Broad Street, Elizabeth, N. J., August 22, 1892.

### INDIANA STATE DENTAL ASSOCIATION.

The Indiana State Dental Association, at its last annual meeting, elected the following officers: President, R. W. Van Valzah. Terre Haute; Vice-President, W. M. Hindman, Vincennes; Secretary, G. E. Hunt, Indianapolis; Treasurer, R. T. Oliver, Indianapolis. The Association will meet in Indianapolis on the last Tuesday in June, 1893.

G. E. Hunt,
*Secretary.*

### CONNECTICUT VALLEY DENTAL SOCIETY.

At the Twenty-seventh Annual Meeting of the Connecticut Valley Dental Society, held at Holyoke, Mass., June 10, 11, and 12, the following officers were elected for the ensuing year: President, Dr. George F. Harwood, of Worcester, Mass.; Vice-Presidents, Dr. F. W. Williams, of Greenfield, Mass., Dr. W. H. Rider, of Danbury, Conn.; Secretary, Dr. George A. Maxfield, of Holyoke, Mass.; Assistant Secretary, Dr. A. J. Cutting, of Southington, Conn.; Treasurer, Dr. W. F. Andrews, of Springfield, Mass.

George A. Maxfield, D.D.S.,
*Secretary.*

### ANNUAL MEETING OF THE ALUMNI ASSOCIATION OF THE NEW YORK COLLEGE OF DENTISTRY.

At the annual meeting of the Alumni Association of the New York College of Dentistry, the following gentlemen were elected to office for 1892:

President, J. Howard Reed; First Vice-President, M. C. Gottschaldt; Second Vice-President, Alfred R. Starr; Secretary, Vincent M. Munier; Treasurer, Zachary T. Sailer.

*Executive Committee.*—Sherman B. Price, H. J. Parker, and E. S. Robinson.

Vincent M. Munier,
*Secretary.*

